# The Doctor’s Wife

**Published:** 1897-03

**Authors:** W. J. Bell


### The Doctor's Wife.

The night was dark and bitter cold,
  The wind across the prairie swept,
While I in comforts warm enrolled
  Snored softly on and soundly slept.
When suddenly my door-bell rang;
  Infernal sound, it pierced my ears,
As on the creaking floor I sprang,
  My heart athrop with direst fears
Lest one had come to call me out
  Into the cruel biting blast.
I for my garments cast about,
  Wishing this night-call were the last.

But oh, the best thought of my life!
  It calms me now as oft before:
I'll send my thoughtful, faithful wife
  To meet the stranger at the door.
She goes, and oh, the sweetest lies
  That ever mortal tongue has told,
As in her artless way she tries
  To say that I'm out in the cold.
"He won't be home till break of day
  And then he'll go, poor, tired man.
I'm awful sorry he's away;
  He'll come as promptly as he can."

I go to bed, but not to sleep;
  I ponder long on doctor's wives,
The only ones who ever think
  Of our rest-broken, weary lives,
I sometimes think God don't record
  Those little white lies often told
To give a way-worn doctor sleep,
  To save him from the winter's cold.
And if He does, I'm sure his pen
  Writes very near, in letters bright,
A tender thought of her who thinks
  Of doctors toiling in the night.

*W. J. Bell, in the American Journalist.*

